# Occupational structure of bearers of Jewish rabbinical, occupational and generic surnames

**DOI:** 10.12688/f1000research.24532.2

**Published:** 2020-10-08

**Authors:** Alexander Jonathan Vidgop, Nelly Norton, Nechama Rosenberg, Malka Haguel-Spitzberg, Itzhak Fouxon

**Affiliations:** 1 Research Department, Am haZikaron Institute for Science and Heritage of the Jewish People, Tel Aviv, 64951, Israel; 2 Department of Mechanical Engineering, Ben-Gurion University of the Negev, Beer Sheva, 84105, Israel

**Keywords:** Occupational Structure, Statistics, Intergenerational Mobility

## Abstract

We study choice of profession in three groups of Russian-speaking Jewish families with different occupational distributions of the ancestors. This study continues exploration of the persistence of social status of families over centuries that was initiated in recent years. It was found previously that in some cases professions remain associated with the same surnames for many generations. Here the studied groups are defined by a class of the surname of individuals composing them. The class serves as a label that indicates a professional bias of the ancestors of the individual. One group are the bearers of the class of surnames which were used by rabbinical dynasties. The other group is constituted by occupational surnames, mostly connected to crafts. Finally, the last group are generic Jewish names defined as surnames belonging to neither of the above groups. We use the self-collected database that consists of 858 and 1057 of the first two groups, respectively, and 7471 generic Jewish surnames. The statistics of the database are those of individuals drawn at random from the considered groups. We determine shares of members of the groups working in a given type of occupations together with the confidence interval. The occupational type’s definition agrees with International Standard Classification of Occupations. It is demonstrated that there is a statistically significant difference in the occupational structure of the three groups that holds beyond the uncertainty allowed by 95% confidence interval. We quantify the difference with a numerical measure of the overlap of professional preferences of different groups. We conclude that in our study the occupational bias of different population groups is preserved at least for two centuries that passed since the considered surnames appeared.

## Introduction

Recently usage of surnames in studies of intergenerational mobility, such as investigations of temporal changes of representations of different surnames in various social groups, has developed into an established tool of research (see e.g. Clark, 2015; Clark, 2012; Clark *et al.,* 2015; Güell *et al.,* 2015 and references therein; Santavirta & Stuhler, 2019 for a recent review). In a typical study, the frequency of occurrence of certain surnames in different professional or elite or other population groups is considered. This frequency is compared with the frequency of the surname’s occurrence in the general population. If it is found that the surname occurs in some group significantly more than in the general population, then the surname is overrepresented in the group. Conversely, if the surname occurs less, then it is underrepresented.

Probably the main result of the aforementioned extensive studies is that the over- or under-representations do not change over long periods of time, much longer than would be implied by the conventional mobility measures. Those measures average over society, thus hiding the underlying low mobility rates for a given surname. These studies have been performed for different countries and cultures (see Clark, 2015 and references therein).

In this work, we perform a study for a population of Russian-speaking Jews who have not been so far considered in this type of study. We investigate occupational distribution of three different groups in the population that are defined by different biases in the occupational distribution of their ancestors. The size of 9315 individuals of the studied pool of data allows us to derive rigorous statistics of the groups (we use here rounding, explained below). We demonstrate that the distributions are different beyond the uncertainty allowed by the confidence interval. This finding shows that biases in occupational distributions can be preserved for at least two centuries that passed, since all Jews have had an inherited surname. This provides yet another demonstration of comparatively slow surnames’ mobility, defined as social mobility of individuals with a given surname, ( Clark, 2015). Our results also provide the occupational distribution of the Russian speaking Jewry of the twentieth century, a result that has its own interest.

The objectives of our study are quite similar to those of Clark (2012), where the statistics of several groups of surnames in Sweden are considered. Clark (2012) considered noble surnames, names that were once given to nobility; Latinized surnames were adopted by the educated class and certain other groups. Similarly, we consider rabbinical surnames (counterpart of nobility), surnames of craftsmen (hereafter called occupational), and others that fall outside of these categories (generic).

Rabbinical surnames are those whose first bearer was a rabbi. Rabbis constituted the elite of their time, the most respected class of the Jewish population, and they can be considered as a kind of the Jewish nobility. The rabbi of the surname’s origin could be a prominent figure living many centuries ago, for example as in the case of Luria, Shapiro or Halperin. Family names of these dynasties were often taken as a sign of distinction. However, other rabbinical families are “only” two hundred years old, for example Rabinovich. For these clans, in the beginning of their history, the profession of the rabbi was often passed from father to son for a number of generations. Occupational surnames derive from the name of the craft of the first bearer of the name. Craftsmen had the knowledge of their craft and present a rough counterpart to the educated class considered by Clark (2012), for example Schuster (shoemaker), Mednik (tinker) and Portnoi (tailor). In these families the professions were also often passed between generations. Finally, generic surnames consist mostly of surnames whose origin has nothing to do with the professions of their founder. Formation of Jewish surnames with few exceptions finished by the beginning of the 19 ^th^ century, see e. g. Beider, 2008.

## Methods

### Participants

Our data was acquired over four years (November 2015 – February 2020) from individuals who were part of an educational family history program that was implemented by the Am haZikaron Institute for Science and Heritage of the Jewish People in Tel Aviv, Israel. Our program was obligatory for participants of a larger, very inclusive program so that to the best of our knowledge the only bias in the sample was some degree of affiliation with the Jewish people.

The individuals were Russian-speaking Jewish family members residing in the Former Soviet Union (FSU). They voluntarily provided genealogical data for the program via online forms that were sent to them before their arrival in Tel Aviv (see section Data collection). The forms were presented in the native language of the individual (Russian) and informed the individuals that their data could be used for future academic purposes. Completion of the form was taken as consent to allow their data to be used for this academic study (some participants chose not to complete the form). This study did not seek ethical approval as it was deemed low risk, none of the participants were considered vulnerable, the participants consented for their data to be used in future academic research, and all participants were over 18 years of age.

Data from an individual was not selected for inclusion in this study if it was intentionally distorted. Data verification was accomplished during a meeting in Tel Aviv between the authors. Upon the completion of the educational program, the participants returned to their home countries.

### Data collection

Information was obtained on professions of a participant’s family members for the last four generations. This resulted in a collection of data on individuals who were born throughout the 20th century. The educational programs at Am haZikaron are open to all, and so there was no known statistical bias toward any particular professions. Similarly, there is no known correlation between the starting letter of the name and the profession. Hence, to the best of our judgement, the obtained data, arranged alphabetically, is a random list of Jewish individuals and their professions. The randomness holds up to small clusters of individuals who belong to the same family and have some correlations. These correlations are yet to be studied and are not the focus of our study. It will be seen later that randomness is consistent with the statistics.

In the research by Clark *et al.* ( Clark, 2015; Clark, 2012; Clark *et al.,* 2015), the major source of information were professional directories that list all individuals in a particular professional area. In contrast, our data is a random pool of the population that necessitates a different methodology for analysis. We partitioned the population into the three groups of surnames (rabbinical, occupational, generic), which could be biased with respect to their occupational distribution due to the bias in their ancestry. We checked if the bias persists through time and found that there is a statistically significant difference in the professional preferences of the three groups.

### Dataset description and considerations

We collected the data over four years until the pool included a statistically significant amount of surnames where the confidence interval allowed to reasonably fix the share of each profession in the total population. We obtained data on 858 (57.8.% men) bearers of rabbinical and 1057 (59.7% men) bearers of occupational surnames. The other 7471 (57.6% men) individuals had a generic surname, which was neither rabbinical not occupational. Men are slightly overrepresented since the maiden names of the female family members were sometimes unknown to the participants. This slight difference in gender composition of the groups may cause some professional bias; however, this is negligible compared with the magnitude of the groups’ differences (shown below).

The studied population included different birth cohorts. The earliest birth date for an individual in the data was 1858 and the latest 2001. We did not perform separate study of different cohorts since the data available for them would not be statistically significant. For adequate comparison of the groups, we must have roughly the same share of each group born in each of the considered generations. Therefore, we divided the historical period spanned by our data into four periods (1858–1894, 1895–1930, 1931–1966, 1967–2001; Table 1).

**Table 1.** Data description showing the percentage of types of surname according to four generations considered.

Generation born	Surname (%)		
	Rabbinical	Occupational	Generic
1858–1894	2.0	2.3	2.2
1895–1930	44.8	46.3	43.7
1931–1966	37.4	35.6	38.0
1967–2001	15.8	15.8	16.1

From Table 1, it is seen that the birth date distributions of the groups are very similar so that the comparison of occupations is reasonable. The difference of shares of different generations holds for many reasons: each participant was asked to fill the data for two parents, four grandparents and eight great grandparents, where the data on the older generations was often forgotten, while the younger generation could still be studying or have no profession yet. However, the precise form of the birth date distribution is irrelevant for our comparative study, for only birth date distributions of different groups are similar.

The birthplaces were scattered all over the territory of the FSU. Jewish families have a long tradition of studying and those who would want to acquire education would typically receive such an opportunity. In other words, with a good approximation, an individual born into a Jewish family of the FSU would have an equal opportunity for getting that or another profession irrespective of birthplace. Therefore, we disregarded the geographical factor in our study.

No detectable bias toward some profession due to a different number of reported family members was observed. This number was never too large, and rarely reached five individuals (other ancestors were not Jewish and not considered by our study).

The data on professions was self-reported in the native (Russian) language of the participant and was not standardized. We processed the data to a standard of occupations according to the methodology below.

### Data analysis

***Grouping the data by profession.*** We separated the data into the three groups described (rabbinical, occupational, generic). We then grouped the professions into 23 narrower professional activities. These were defined either by their significant presence in the data, e.g. bookkeepers who constituted about 5% of all individuals, or by a unique character of the profession, for example interpreter/linguist. The groupings of professions, when performed, did not contradict the International Standard Classification of Occupations. The 23 categories of professions were as follows:

1. Engineer – by far the largest fraction of the studied population
2. Physician
3. Teacher
4. Bookkeeper
5. Worker
6. Creative profession
7. Economist
8. Head/chief officer
9. Nurse
10. Researcher
11. Clerical worker
12. Armed forces
13. Programmer
14. Salesmen
15. Businessman
16. Legal professional
17. Driver
18. Interpreter, linguist
19. Literary worker
20. Pharmacist
21. Librarian
22. Psychologist
23. Rarely occurring profession (other)

We calculated the number of individuals having each one of the above professions for each of the three studied groups of surnames. The main target of our study is the share of each profession (P _i_) in each of the considered three groups of surnames. Thus, if N is the total number of members of Russian-speaking Jewish families with a generic surname then P _i_*N is the total number of members of these families with the i-th profession. For instance, P _21_*N would be the total number of librarians. The data on the full group consisting of millions of people (as defined by the fraction of Russian-speaking Jews whose names are neither rabbinical nor occupational where we count not only our contemporaries but all those who lived in the twentieth century) are unavailable. Thus, we have the standard problem of constraining P _i_ from the incomplete information on the studied groups that is at our disposal. This is done by the statistical analysis relying on the observation that with good approximation our data constitute a random pool of the considered population groups.

A typical result of counting the professions is presented in Table 2 where the example of generic surnames is used. The total pool of data consisted of 7471 individuals. Due to presence of correlated clusters of individuals in the data, we found it instructive to use coarse-grained variables X _i_(k) for the statistical calculations. These variables separate the data into blocks of hundreds. In contrast with individual data which is not randomly sampled, the blocks can be be considered as a result of random sampling where a hundred was taken from the population and then, independently, another hundred and so forth, see below. Moreover separation into blocks demonstrates what we can anticipate to see if we pick 100 members from the considered population: for the pool size of 100, the statistical properties are seen already. Frequencies of different professions in each block of 100 are similar with some fluctuations. The usage of the variables X _i_(k) is necessary for the statistical considerations as explained in detail below, otherwise they give an idea how the described laws apply in practice when the sample sizes are moderate. The statistics of X _i_(k) answers the question – if we took a pool of 100 representatives of one of the groups what would be the typical occurrences of each profession? 

**Table 2.** Results of counting the number of individuals with different professions by hundreds for the pool of 7471 bearers of generic surnames. The first column provides the profession. The entries of the first row provide the considered range of numbers of people in the list considered in the corresponding column. For instance, the second column describes occupational distribution of 100 individuals with numbers from 1 to 100, the third column describes 100 individuals with numbers from 101 to 200 and so forth. Thus X _7_(300) is the number of individuals with the profession of economist in the range from 201 to 300. This number is located at the intersection of eighth row and fourth column, X _7_(300)=2 – our list contains two economists in the portion of the list defined by 201–300 range.

Occupations	1–100	101–200	201–300	301–400	401–500	501–600	601–700	701–800	801–900	901–1000	Mean, 7471
1. Engineer	18	17	23	15	15	17	22	21	13	21	19.8
2. Physician	5	8	7	10	11	10	10	3	5	8	7.4
3. Teacher	12	7	7	10	10	13	12	5	11	8	9.8
4. Bookkeeper	6	6	5	3	1	4	1	4	4	9	4.9
5. Worker	13	15	16	21	20	17	10	21	22	18	18.6
6. Creative profession	8	6	5	6	7	3	5	3	8	2	4
7. Economist	2	3	2	3	1	4	3	6	5	3	3.1
8. Head/chief officer	3	3	1	2	3	6	4	1	8	4	3.6
9. Nurse	5	4	7	4	1	5	2	3	1	1	2.9
10. Researcher	1	3	4	3	5	2	9	6	0	1	3.7
11. Clerical worker	3	5	4	4	8	3	3	3	5	7	4.5
12. Armed forces	4	1	2	1	5	3	4	6	5	4	3.7
13. Programmer	2	2	4	1	2	0	1	1	0	1	0.8
14. Salesman	9	6	4	5	5	4	3	1	3	4	3.5
15. Businessman	5	8	2	5	1	3	1	5	4	2	2.9
16. Legal profession	1	0	0	1	1	1	1	5	1	1	1.8
17. Driver	0	1	1	2	0	2	2	3	1	1	1.6
18. Interpreter, linguist	0	0	1	1	0	0	1	0	1	0	0.3
19. Literary worker	2	1	0	1	1	0	0	0	1	1	0.6
20. Pharmacist	0	1	3	0	0	1	4	1	0	1	0.9
21. Librarian	0	1	1	1	0	1	1	0	0	1	0.4
22. Psychologist	0	0	1	1	0	0	0	0	1	2	0.5
23. Other	1	2	0	0	3	1	1	2	1	0	0.9

Thus we took out of our pool the first 100 individuals and determined the numbers X _i_(100) of individuals with the i-th profession. Then we took 100 more individuals and determined the numbers X _i_(200) of individuals with the i-th profession in the range of 101–200. Continuing in this way, we determined X _i_(k) determined by the columns in Table 2. We used the pool size up to 1000 because this size allows comparison with the groups of rabbinical and occupational surnames where the total pool was about 1000. Thus, we used the pool of 7471 as the control size that allows to test how well the pool of 1000 individuals represents the whole considered group. Average over a limited pool size k gives an approximation p _i_(k) for P _i_ defined above. Up to fluctuations p _i_(k) monotonously approach P _i_ on increasing k and we can hope that a reasonable approximation to P _i_ can be obtained already from the largest k available from our data. Indeed, we demonstrate quantitatively that distributions derived from the pools of 1000 and 7471 individuals are rather similar. Thus, making the reasonable assumption that pool size of 7471 represents the full group accurately, which is proved by the calculation of confidence intervals, we conclude that the occupational distribution of the generic surnames can be derived quite accurately (quantified below) from the distribution of 1000 individuals. Assuming that the groups of bearers of rabbinical and occupational surnames are similar statistically we can then conclude that the distributions for these groups, derived from the study of about 1000 individuals, provide a good characterization of the full groups. Despite that, we rely in our conclusions on the rigorously defined confidence intervals; qualitatively it is probable that the means obtained from studies of 858 rabbinical and 1057 occupational surnames provide accurate idea of the full groups.

**Figure 1.**
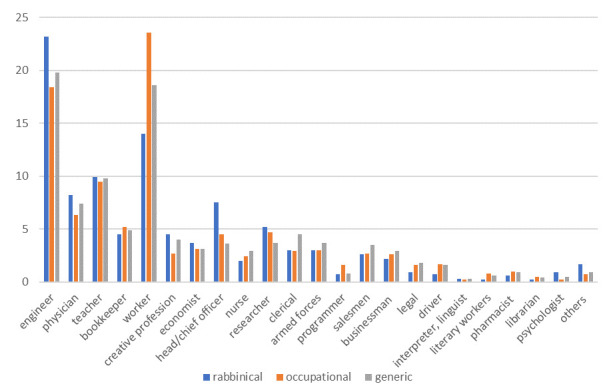
Occupational distributions of the three groups of surnames according to the 23 professional activities (%).

***Distance between the distributions.*** There was a need for quantitative comparison of distributions P _i_ of the three considered groups ( Figure 1). Thus, considering the finite pool sizes’ approximations to P _i_ in Figure 1 (in %), it is seen that they are different; however how different? To make the comparison, we calculated the distance between the distributions. There is no unique conventional definition of the distance between probability distributions. We used the Hellinger distance (see for example. Yang & Le Cam, 2000), whose definition can be understood by observing that since the distributions are normalized, $\sum_{i=1}^{23}{\text{P}}_{i}=1,$ then what needs the comparison are the shapes of the distributions. For instance, for all three distributions in Figure 1, the heights of all bars sum to 100 and only the shapes distinguish the distributions. The shapes can be compared by considering the overlap, which is conveniently defined by the Bhattacharyya coefficient ( Bhattacharyya, 1943):

$$\text{BC}(\text{k},\;\text{p})\equiv \sum_{\text{i}=1}^{23}\sqrt{{\text{P}}_{\text{i}}^{\text{k}}{\text{P}}_{\text{i}}^{\text{p}}},$$

where k and p are indices of the considered two groups (in this equation and below); when there is a need to indicate to which group P _i_ refers, we use the notation ${\text{P}}_{\text{i}}^{\text{r}},$ ${\text{P}}_{\text{i}}^{\text{o}},$ ${\text{P}}_{\text{i}}^{\text{g}},$ for the P _i_ of rabbinical, occupational and generic surnames, respectively. The Bhattacharyya coefficient is the scalar product (type of overlap) of two vectors in 23-dimensional space with components $\sqrt{{\text{P}}_{\text{i}}^{\text{k}}}$ and $\sqrt{{\text{P}}_{\text{i}}^{\text{p}}}$. The square root is introduced in the definition because $\sqrt{{\text{P}}_{\text{i}}^{\text{k}}}$ are unit vectors in the 23-dimensional space, which allows definition of “the shape of the distribution” as the direction of these unit vectors. The coefficient changes between zero, holding for non-overlapping distributions, and one, holding for identical distributions. The Hellinger distance between the distributions is then defined as:

$$\text{H}(\text{k},\;\text{p})\equiv \sqrt{1-\text{BC}(\text{k},\;\text{p})}=(1/\sqrt{2})\sum_{i=1}^{23}(\sqrt{{\text{P}}_{\text{i}}^{\text{k}}}-\sqrt{{\text{P}}_{\text{i}}^{\text{p}}}{)}^{2}.$$

Thus, H(k, p) is proportional to the Euclidean distance between two vectors $\sqrt{{\text{P}}_{\text{i}}^{\text{k}}}$ and $\sqrt{{\text{P}}_{\text{i}}^{\text{p}}}$ in the 23 dimensional spaces. It provides a good definition of the distance because the Euclidean distance does. We will see below that on their own the distances do not allow the distributions’ comparison, however in conjunction with statistical analysis they become useful.

***Confidence interval under the random sampling assumption*.** Our sample consists of 858 bearers of rabbinical surnames, 1057 bearers of occupational surnames and 7471 bearers of generic surnames (we often use in the calculations the rounded number of 7400). These samples are quite large; however, the population means that can be derived from them still contain quite a large uncertainty. Here we describe the derivation of the confidence interval, i.e. the interval within which the population means are contained with high probability (95% probability in our study). The derivation in this section is done assuming that our list of individuals is a random sample of the studied population groups. This is a good assumption up to the presence of sequences of 3–5 individuals with the same surname who belong to the same close family. These individuals can be assumed to have a certain correlation of professions, which violates the assumption of random sampling. The consistency of the assumption of random sampling despite these correlations will be demonstrated in the next section.

The material in the rest of this section is mostly well-known. We consider a total population of X individuals, of whom X _i_ have a property “i”. In the application of interest in this work, this property is a certain profession; however, the nature of this property is irrelevant for general considerations. We make a random sampling of the population, i.e. we pick an individual at random. Then, by definition of random sampling, the individual with property “i” is picked with probability p _i_ = X _i_/X. If we continue the random sampling, then the probability distribution of the number X _i_(N) of individuals with the property “i” in randomly picked N individuals is given by the binomial distribution with the success probability p _i_. Here we assume that N is much smaller than both X _i_ and X so that the random sampling occurs in approximately identical conditions. The average and variance of X _i_(N) are given by the well-known formulas of binomial distribution

$$<{\text{X}}_{\text{i}}(\text{N})>={\text{p}}_{\text{i}}\;\text{N},\;<{\left({\text{X}}_{\text{i}}(\text{N})-<{\text{X}}_{\text{i}}(\text{N})>\right)}^{2}>={\text{p}}_{\text{i}}\;\text{N}(1-{\text{p}}_{\text{i}}),\;(1)$$

where here and below the angular brackets stand for averaging. Large N binomial distribution can be approximated by Gaussian, implying that the distribution of X _i_(N) is Gaussian and is determined uniquely by the mean and the variance above. We find that the distribution of x≡(X _i_(N)- p _i_ N)/[p _i_ N(1- p _i_)] ^1/2^ is the standard normal distribution with zero mean and unit variance. For this distribution, it is well-known that the probability that x will fall between -1.96 and 1.96 is approximately 0.95. This probability equals the probability that X _i_(N)/N falls between p _i_-[p _i_(1- p _i_)/N] ^1/2^ *1.96 and p _i_+[p _i_(1- p _i_)/N] ^1/2^ *1.96 that is designated by $\text{P}({\text{p}}_{\text{i}}-1.96*\sqrt{{\text{p}}_{\text{i}}(1-{\text{p}}_{\text{i}})/\text{N}}\leq {\text{X}}_{\text{i}}(\text{N})/\text{N}\leq {\text{p}}_{\text{i}}+1.96*\sqrt{{\text{p}}_{\text{i}}(1-{\text{p}}_{\text{i}})/\text{N}})$ and obeys

$$\text{P}({\text{p}}_{\text{i}}-1.96*\sqrt{{\text{p}}_{\text{i}}(1-{\text{p}}_{\text{i}})/\text{N}}\leq {\text{X}}_{\text{i}}(\text{N})/\text{N}\leq {\text{p}}_{\text{i}}+1.96*\sqrt{{\text{p}}_{\text{i}}(1-{\text{p}}_{\text{i}})/\text{N}})=0.95.\;(2)$$

Our data provides X _i_(N)/N, from which we want to find the confidence interval of p _i _that is the interval to which p _i _belongs with 95% certainty. If the observation provides the value X _obs_ for X _i_(N) then, with 95% probability, the unknown quantity p _i _obeys

$$-1.96\leq ({\text{X}}_{\text{obs}}-{\text{p}}_{\text{i}}\;\text{N})/\sqrt{{\text{p}}_{\text{i}}\;\text{N}(1-{\text{p}}_{\text{i}})}\leq 1.96.$$

This inequality is equivalent to (X _obs_ - p _i_ N) ^2^ ≤1.96 ^2^ p _i_ N(1- p _i_), which gives

$${\text{p}}_{\text{i}}^{\text{2}}\;({\text{N}}^{2}+1.96^{2}\;\text{N})-{\text{p}}_{\text{i}}\;{\text{N(2X}}_{\text{obs}}+\text{1}{\text{.96}}^{\text{2}}\text{)}+{\left({\text{X}}_{\text{obs}}\right)}^{2}\leq 0.$$

We find

$${\text{(X}}_{\text{obs}}+\text{1}{\text{.96}}^{\text{2}}\text{/2)}/(\text{N}+1.96^{2})-\text{Y}\leq {\text{p}}_{\text{i}}\leq ({\text{X}}_{\text{obs}}+1.96^{2}/2)/(\text{N}+1.96^{2})+\text{Y},$$

where we defined

$$\text{Y}\equiv \sqrt{{\text{N}}^{2}\;{{\text{(2X}}_{\text{obs}}+\text{1}{\text{.96}}^{\text{2}}\text{)}}^{\text{2}}-4\;{{\text{(X}}_{\text{obs}})}^{\text{2}}\;{\text{(N}}^{\text{2}}+\text{1}{\text{.96}}^{\text{2}}\;\;\text{N)}}/2{\text{(N}}^{\text{2}}+\text{1}.96^{\text{2}}\;\text{N)}.$$

We have neglected terms of order 1/N and introducing (pi) _obs_≡ X _obs_/N that

$${\left({\text{p}}_{\text{i}}\right)}_{\text{obs}}-1.96*\sqrt{{\left({\text{p}}_{\text{i}}\right)}_{\text{obs}}\;(1-{\left({\text{p}}_{\text{i}}\right)}_{\text{obs}})/\text{N}}\leq {\text{p}}_{\text{i}}\leq {\left({\text{p}}_{\text{i}}\right)}_{\text{obs}}+1.96*\sqrt{{\left({\text{p}}_{\text{i}}\right)}_{\text{obs}}(1-{\left({\text{p}}_{\text{i}}\right)}_{\text{obs}})/\text{N}},$$

with 95% probability. This has the same form as Equation (2) above because p _i _and (p _i_) _obs_ coincide in the leading order in N>>1. For not so large sample, however, there is a difference, the property which is often not mentioned in the discussions.

In the rest of this work we keep using the definition of the confidence interval by 95% certainty. This and the factor of 1.96 above are somewhat arbitrary. If we used 90% confidence interval instead, then 1.645 would be present in the formula above instead of 1.96. This would result in more differences between the studied groups; however, we prefer to stick to the more conservative estimate of the differences.

***Data consistency with random sampling.*** We have already reported that the assumption of perfectly random sampling is not accurate. However, our data is still composed of independent information units where the unit is defined by the information provided by one participant. This unit is the information on the close family of the participant. Hence for a large number of participants, considering with very good approximation the information that they provide as independent, the data is still the sum of independent identically distributed random variables. The variable is the number of people with profession “i” in one reported family. Therefore, by the central limit theorem (see e.g. Gnedenko & Kolmogorov, 1968), the distribution of the number of representatives of each profession is still Gaussian, as in the case of random sampling. Having the Gaussian distribution, we can then evaluate the confidence interval and obtain the appropriate generalization of the results of the previous section. Below we quantify these considerations and provide the changes that are in order.

Table 3 provides a typical excerpt from our list of individuals and their professions. It is seen that some surnames are included multiple times. In Table 3, individuals with the same surname do not necessarily belong to the same family, as seen from the places of origin. In some cases (not shown), the bearers of the same names did belong to the same family and had similar professions. For instance in the list of 858 individuals with rabbinical surnames, we found two Berlins who were architects, two Wahls who were salesmen, two Halperins who were accountants, two Hellers who were teachers, three Hellers who were engineers, four Hellers who were economists, two Ginzburgs who were accountants and two who worked in delivery, two Gordovers who were engineers, two Gordons who were workers, and nine Horowitzs who were engineers. These correlations might cause the statistics to behave differently from the situation where each individual is picked at random from the studied group. This issue must be considered quantitatively. We observed previously that the number of reported people from the same family rarely exceeded five and typically was much less. For quantitative treatment of the effect of these correlations of limited range (below five), we consider the statistics of X _i_(N) – the number of individuals with profession “i” in the list with total number of individuals N. We introduce the random variable x _i_(p). This variable equals 1 if the individual in place “p” of the list has profession “i” and 0 otherwise. Then

$${\text{X}}_{\text{i}}(\text{N})=\sum_{\text{p=1}}^{\text{N}}{\text{x}}_{\text{i}}(\text{p}).$$

**Table 3.** Excerpt from the list of surnames with rabbinical origin. Some surnames are different spellings of the same name. These spellings were created in the course of family migrations during centuries because spellings of the same name in official documents differed in different countries, for example due to spelling mistakes in the records.

Rabbinical surnames	Place of birth	Profession
Eisenstadt	Lithuania	Forwarding miner
Axelrod	Nevel, Belarus	Paramedic
Axelrud	Kiev, Ukraine	Military
Alexandrov	Minsk, Belarus	Engineer
Alexandrovich	Olgopol, Ukraine	Teacher
Aleshin (Epstein)	Kiev, USSR	Teacher
Alpern	Kharkov, Russia	Statistician
Alperovich	Minsk, Belarus	Civil engineer
Altshuler	Katav-Ivanovsk, Russia	Geologist, physicist
Altshuler	Bialynichy, Belarus	Tailor
Altshuler	Unknown	Rabbi
Altshuller	Zhmerynka, Ukraine	Engineer
Amdur	Odessa, Ukraine	Seismologist
Amdursky	Bialystok, Poland	Employee
Ashkenazi	Odessa, Ukraine	Composer
Baalshem	Balta, Ukraine	Worker
Bachrach	Vitebsk, Belarus	Head of tobacco factory
Berlin	Moscow, Russia	Architect
Berlin	Omsk, Russia	Historian/military
Berlin	Odessa, Ukraine	Architect
Bloch	Dzerzhinsk, Ukraine	Accountant

We assume that the list is ordered so that individuals with possible correlations of professions are grouped together; the list can be thought of as obtained in this way. We pick at random individuals from the considered group (bearers of rabbinical or occupational or generic surname) and then we pick their close family of random size as determined by the statistics of family sizes (which is of no interest here). This implies that x _i_(p) in the equation above have finite correlation range so that the variance < x _i_(p) x _i_(p+k)>- < x _i_(p) > < x _i_(p+k)> is non-zero for some positive integer k. k is bounded from above by k _max_, which is formally given by the maximal family size, which is fifteen (individual, parents, great and great great-parents); however, this is five in reality as we have already explained. For large N>>k _max_ the distribution of X _i_(N) is still Gaussian as in the case of random sampling. This can be seen as the result of application of the central limit theorem to the sum of independent random variables where each variable is the sum of x _i_(p) over one family, i.e.

$${\text{X}}_{\text{i}}(\text{N})=\sum_{\text{l}}^{}{\left({\text{y}}_{\text{family}}\right)}_{\text{l}},\;{\left({\text{y}}_{\text{family}}\right)}_{\text{l}}=\sum_{\text{l-th family}}{\text{x}}_{\text{i}}\text{(p)}.$$

Here we introduced the random variable (y _family_) _l _, which counts the number of representatives of profession “i” in the l-th family where l is the index of the family. Thus the statistics of (y _family_) _l _could be obtained by partitioning the considered group of population (rabbinical, occupational or generic) into families, where the family is defined as information unit in our data and not otherwise, with the unit’s definition given in the beginning of this section. Considering (y _family_) _l _with different l as independent, we conclude from the central limit theorem that X _i_(N) is approximately Gaussian random variable, which is uniquely characterized by its mean and variance (a finer consideration can be done using the version of the central limit theorem for random variables with fast decaying correlations, see Gnedenko and Kolmogorov). These are given by

$${\text{< X}}_{\text{i}}{\text{(N)}\;>=\text{p}}_{\text{i}}\;{\text{N},\;<\text{(X}}_{\text{i}}{\text{(N)-<X}}_{\text{i}}{\text{(N)}\;>\text{)}}^{\text{2}}\text{>= }\sum_{\text{p=1}}^{\text{N}}\;\sum_{\text{k=1}}^{\text{N}}{\text{(<x}}_{\text{i}}{\text{(p)x}}_{\text{i}}{\text{(k)>- <x}}_{\text{i}}{\text{(p)}>\;<x}_{\text{i}}\text{(k)>)}\;(3)$$

where we used directly the definition X _i_(N)= ∑ ^N^_p=1_ x _i_(p). Here <x _i_(p)>= p _i_, where p _i_ were defined in the previous section. We can consider the last sum as sum of diagonal and off-diagonal elements. The sum over the diagonal elements is readily found by using that x _i_(p)=1 with probability p _i_ and x _i_(p)=0 with probability 1-p _i_. Thus <x ^2^_i_(p)>=p _i_ and

$$\sum_{\text{p=1}}^{\text{N}}{\text{(<x}}_{\text{i}}^{\text{2}}{\text{(p)> -<x}}_{\text{i}}{\text{(p)>}}^{\text{2}}{\text{)=p}}_{\text{i}}\;{\text{N(1-p}}_{\text{i}}\text{),}$$

which is the same answer as for the random sampling. It is the sum of the off-diagonal terms ∑ _p≠k_ (<x _i_(p) x _i_(k)>- <x _i_(p) > <x _i_(k)>), a non-trivial quantity, which characterizes the correlations of professions within one family. Thus <x _i_(p) x _i_(p+1)> is the probability that two consecutive individuals from the list have identical profession “i”. This probability is larger than <x _i_(p)> <x _i_(p+1)>, which would hold if professions of the individuals were independent. The finite difference <x _i_(p) x _i_(p+1)>- <x _i_(p)> <x _i_(p+1)> is caused by the finite probability that individuals p and p+1 belong to the same family and thus have positively correlated professions. It seems inevitable that within-family correlations are positive so that

$${\text{<x}}_{\text{i}}{\text{(p) x}}_{\text{i}}{\text{(p+k)> - <x}}_{\text{i}}{\text{(p)><x}}_{\text{i}}\text{(k)>}\geq \text{0}.$$

The left-hand side of the above equation is identically zero for k> k _max_ (see above). It is then readily seen from the definition in Equation (3) above that the variance obeys

$$<{\left({\text{X}}_{\text{i}}(\text{N})\;\text{ - <}{\text{X}}_{\text{i}}(\text{N})>\right)}^{2}\text{>=}\text{<(}{\text{X}}_{\text{i}}{\text{(N))}}^{\text{2}}\text{>}-<{\text{X}}_{\text{i}}(\text{N}){>}^{2}={\text{c}}_{\text{i}}\;\text{N}\;\text{N}>>{\text{k}}_{\text{max}}$$

where the constant c _i_ is independent of N. The question is how different c _i_ is from the random sampling value p _i_(1-p _i_) because of the described correlations.

We face the problem of estimating the normalized dispersion c _i_ for our unknown sampling statistics. This can be done quite accurately in the case of bearers of generic surnames where we have data on more than 7400 individuals. This is accomplished by partitioning the list into 74 hundreds and considering the corresponding 74 numbers [X _i_(100)] ^k^ with k running from 1 to 74 as independent realizations of X _i_(100). Here the independence of [X _i_(100)] ^k^ with different k holds due to 100>>k _max_, which implies that the number of correlated professions in different hundreds is negligibly small in comparison with the total numbers. Indeed, we have

$${\text{<[X}}_{\text{i}}\text{(100)]}{\;}^{\text{k}}{\text{[X}}_{\text{i}}\text{(100)]}{\;}^{\text{k+1}}\text{>=<(}\sum_{\text{p=1}}^{\text{100}}{\text{x}}_{\text{i}}\text{(p)) (}\sum_{\text{k=101}}^{\text{200}}{\text{x}}_{\text{i}}\text{(k))> =}\sum_{\text{p=1}}^{\text{100}}\sum_{\text{k=101}}^{\text{200}}{\text{<x}}_{\text{i}}{\text{(p)x}}_{\text{i}}\text{(k)>}$$

where <x _i_(p)(x _i_(k)> differs from <x _i_(p)><x _i_(k)> only for indices p and k in narrow vicinity of p=k=100, which can be neglected. We find the independent variables law <[X _i_(100)] ^k^[X _i_(100)] ^k+1^>=<[X _i_(100)] ^k^><[X _i_(100)] ^k+1^> (similar consideration can be done for higher order correlations). This independence is the reason why we group the data in blocks of 100 (of course the block size is not defined uniquely). We observe that Y _i_≡(1/74) ∑ ^74^_k=1_([X _i_(100)] ^k^) ^2 ^is a sum of independent Gaussian variables and hence it is Gaussian itself. The average of Y _i_ is <(X _i_(100)) ^2^> and its dispersion is

$${\text{<Y}}_{\text{i}}^{\text{2}}{\text{> - <Y}}_{\text{i}}{\text{>}}^{\text{2}}{\text{=(1/74}}^{\text{2}}\text{)}\;\sum_{\text{k=1}}^{\text{74}}{\text{(<([X}}_{\text{i}}\text{(100)]}{\;}^{\text{k}}{\text{)}}^{\text{4}}\text{>}{\;}^{\text{-}}\;{\text{<([X}}_{\text{i}}{\text{(100)]}}^{\text{k}}{\text{)}}^{\text{2}}{\text{>}}^{\text{2}}\text{)}\;{\text{=(1/37) <(X}}_{\text{i}}{\text{(100)])}}^{\text{2}}{\text{>}}^{\text{2}}.$$

where we used independence of [X _i_(100)] ^k^ with different k and that Gaussianity of [X _i_(100)] ^k^ implies <([X _i_(100)] ^k^) ^4^>=3 <([X _i_(100)] ^k^) ^2^> ^2^=3 <(X _i_(100)]) ^2^> ^2^. Thus the distribution of (Y _i_/<(X _i_(100)) ^2^>-1) √37 is the standard normal distribution with zero mean and unit variance. We find that the value of Y _i_ obtained from our data limits <(X _i_(100)) ^2^> within the interval given by

$${\text{Y}}_{\text{i}}\;-1{\text{.96Y}}_{\text{i}}\text{/}\sqrt{37}{\text{<<(X}}_{\text{i}}{\text{(100))}}^{\text{2}}\text{>}\;\text{<}\;{\text{Y}}_{\text{i}}\text{+1}{\text{.96 Y}}_{\text{i}}\text{/}\sqrt{37}$$

with the confidence level of 95% (see above). The random sampling would give <(X _i_(N)) ^2^>= p _i _N+N(N-1) p _i_^2^ with N=100 as seen readily from the properties of the binomial distribution. The comparison between the dispersion determined from our data and the dispersion predicted by the random sampling assumption is provided in Table 4. 

**Table 4.** Comparison of dispersion evaluated from the sample data (second column) and the random sampling prediction (third column) for generic surnames. The agreement is found to be much narrower than allowed by the confidence interval provided in the fourth and fifth columns.

Occupations	Observed <(X _i_(100)) ^2^>; Σ ^74^_k=1_([X _i_(100)] ^k^) ^2^/74	Random sampling; <(X _i_(N)) ^2^>=100 p _i_ +9900p _i_^2^	Lower end of confidence interval	Upper end of confidence interval
1. Engineer	410.6	407.8	278.2	542.8
2. Physician	61.3	61.1	41.6	81.1
3. Teacher	104.6	104.3	70.9	138.2
4. Bookkeeper	28.4	28.2	19.2	37.5
5. Worker	361.4	359.4	245	477.8
6. Creative profession	20.6	19.5	13.9	27.2
7. Economist	12.5	12.6	8.5	16.5
8. Head/chief officer	17.9	16.5	12.1	23.6
9. Nurse	11.8	10.9	8	15.6
10. Researcher	17.9	17.4	12.1	23.6
11. Clerical worker	26.1	24.7	17.7	34.6
12. Armed forces	17.5	17.6	11.9	23.2
13. Programmer	1.4	1.4	0.9	1.8
14. Salesman	16.2	15.8	11	21.5
15. Businessman	11.5	11.5	7.8	15.1
16. Legal profession	5.5	4.8	3.7	7.3
17. Driver	4.4	4.2	3	5.8
18. Interpreter, linguist	0.3	0.3	0.2	0.4
19. Literary worker	0.9	0.9	0.6	1.2
20. Pharmacist	1.9	1.6	1.3	2.5
21. Librarian	0.5	0.5	0.4	0.7
22. Psychologist	0.7	0.8	0.5	0.9
23. Other	1.9	1.7	1.3	2.5

We find that the observed dispersion agrees with the prediction of the random sampling assumption with accuracy which is well beyond what could be hoped for, as is clear from the confidence interval. Here we used for p _i_ the values obtained from averaging over the sample of more than 7400 individuals, where we neglect the error using the large sample size. The observed agreement over as many as 23 categories is completely consistent with the assumption that the data are equivalent to random sampling of the group of bearers of generic surnames. In other words, the correlations between the profession of different individuals, which are present in the data, are negligible. Since these correlations do not seem to be different for other groups (bearers of rabbinical and artisanal surnames) then we will assume in the Results below that our data provides the random sampling of all the considered population groups. We have also derived dispersion for other groups and saw that the assumption works well (these comparisons are not provided since for these groups the sample of about 1000 individuals is too small for reaching rigorous conclusions). 

## Results

The results for the occupational distribution of the generic surnames derived from data presented in Table 2 are given in Table 5. For this surnames’ class we have a rather large pool, which allows us to obtain a distribution with high accuracy, as presented in the third column, which gives the mean together with the confidence interval. It is seen that the means are fixed rather sharply and for many categories the range of values around the mean, allowed by the confidence interval, is narrow. Yet sharper results hold for the total pool of 9315 individuals consisting of all the surnames, i.e. the generic, rabbinical and occupational surnames together (here 9315 is found using the data on only 7400 out of 7471 individuals with generic surname). The distribution, presented in the fourth column, provides us with rather detailed information on the occupations of the Russian-speaking Jews that seemingly was not considered previously. Finally, the second column presents the same distribution, however obtained by restricting the pool of generic surnames to the first 1000 individuals. This distribution is provided for comparison with rabbinical and occupational surnames in Table 6 where the total available pool is about 1000 in both cases.

**Table 5.** Occupational distribution of generic and all surnames. The third column provides population means together with the confidence interval as derived from the full pool of 7400 individuals. For comparison with other surnames’ classes, we present in the second column also the distribution that would be derived by using only the first 1000 names.

Occupations	Generic surnames confidence interval, % (n=1000)	Generic surnames confidence interval, % (n=7400)	All surnames confidence interval, % (n=9315)
1. Engineer	18.2±2.4	19.8±0.9	20±0.8
2. Physician	7.7±1.7	7.4±0.6	7.3±0.5
3. Teacher	9.5±1.8	9.8±0.7	9.7±0.6
4. Bookkeeper	4.3±1.3	4.9±0.5	4.9±0.4
5. Worker	17.3±2.3	18.6±0.9	18.7±0.8
6. Creative profession	5.3±1.4	4±0.4	3.9±0.4
7. Economist	3.2±1.1	3.1±0.4	3.2±0.4
8. Head/chief officer	3.5±1.1	3.6±0.4	4.1±0.4
9. Nurse	3.3±1.1	2.9±0.4	2.7±0.3
10. Researcher	3.4±1.1	3.7±0.4	4±0.4
11. Clerical worker	4.5±1.3	4.5±0.5	4.2±0.4
12. Armed forces	3.5±1.1	3.7±0.4	3.6±0.4
13. Programmer	1.4±0.7	0.8±0.2	0.9±0.2
14. Salesman	4.4±1.3	3.5±0.4	3.3±0.4
15. Businessman	3.6±1.2	2.9±0.4	2.8±0.3
16. Legal profession	1.2±0.7	1.8±0.3	1.7±0.3
17. Driver	1.3±0.7	1.6±0.3	1.5±0.2
18. Interpreter, linguist	0.4±0.4	0.3±0.1	0.3±0.1
19. Literary worker	0.7±0.5	0.6±0.2	0.6±0.2
20. Pharmacist	1.1±0.6	0.9±0.2	0.9±0.2
21. Librarian	0.6±0.5	0.4±0.1	0.4±0.1
22. Psychologist	0.5±0.4	0.5±0.2	0.5±0.1
23. Other	1.1±0.6	0.9±0.2	1±0.2

**Table 6.** Occupational distributions for different sections of the considered group of Russian-speaking Jews, only means shown. The second column provides the distribution of the generic surnames (considered previously), the third gives the distribution for the joined classes of generic and rabbinical surnames and the fourth for generic and occupational surnames.

Occupations	Generic surnames (n=7400)	Generic (n=7400) + Rabbinical (n=858) surnames	Generic (n=7400) + Occupational (n=1057) surnames
1. Engineer	19.8	20.2	19.5
2. Physician	7.4	7.4	7.2
3. Teacher	9.8	9.8	9.7
4. Bookkeeper	4.9	4.8	4.9
5. Worker	18.6	18.1	19.1
6. Creative profession	4	4	3.8
7. Economist	3.1	3.2	3.1
8. Head/chief officer	3.6	4	3.7
9. Nurse	2.9	2.8	2.8
10. Researcher	3.7	3.9	3.8
11. Clerical worker	4.5	4.4	4.3
12. Armed forces	3.7	3.7	3.6
13. Programmer	0.8	0.8	0.9
14. Salesman	3.5	3.4	3.4
15. Businessman	2.9	2.9	2.9
16. Legal profession	1.8	1.7	1.7
17. Driver	1.6	1.5	1.6
18. Interpreter, linguist	0.3	0.3	0.2
19. Literary worker	0.6	0.5	0.6
20. Pharmacist	0.9	0.8	0.9
21. Librarian	0.4	0.4	0.4
22. Psychologist	0.5	0.6	0.5
23. Other	0.9	1	0.9

We observe that the distributions of bearers of generic surnames and of the total considered population are rather similar. In fact, within the confidence interval, the distributions agree (we observe however quite significant difference in the predicted means in the rows marked with blue color). The Bhattacharyya coefficient of these distributions is 0.9998 and the Hellinger distance between the distributions is 0.014 (the calculation demands the full and not rounded numbers). The coefficient is very close to one and the distance is very small; however, the interpretation of these numbers is not obvious. We need a scale to tell which distance is large and which small because the vectors representing the distributions belong to a high-dimensional space. For comparison, the coefficient and the distance for the distributions of the generic surnames in the second and third columns of the table are 0.9975 and 0.05 respectively. These numbers are also very close to one or small despite the difference of the distributions being quite appreciable (the difficulty in introducing measures of similarity in high dimensional spaces is sometimes known as “the dimensionality curse”; see e.g. Aggarwal *et al.,* 2001).

Thus, we compare the distributions of generic and all surnames directly ( Table 5). The distributions, as given by the mean values, are very similar. We marked in blue the only three rows for which the distributions differ appreciably where the maximal difference is 12%. The distributions’ difference is insignificant both because the share of the rabbinical and occupational surnames in the total population is not that large (~20%), and the difference of the distributions of the generic, rabbinical and occupational surnames is not very large. Yet the difference exists, and it is statistically significant as we will demonstrate.

The lack of the appreciable difference between the total distribution and the generic distribution has the origin that is similar to that of measured high mobility rates in different countries. These measurements do not contradict low mobility rates measured by surname, as explained in Clark, 2015. Different surnames are over- or under-represented in different social groups for long periods of time; however, when society’s average is taken for deriving the mobility rate of society, the over- and under-representation average out producing overall high mobility rates. Similarly, the deviations of the rabbinical and occupational surnames’ distributions from that of the generic surnames often occur in opposite directions, so that after averaging the difference disappears. This point is illustrated in Table 6. We see that for the largest absolute deviations, marked in red, the deviations are opposite, including the row corresponding to head/chief officer. Indeed, for this row, the deviations of the generic and all surnames are strongest. 

Furthermore, we see from Table 5 that population means predicted from the study of 1000 and 7400 individuals are consistent within the confidence interval. Moreover, the average values coincide with high accuracy – for 13 occupational categories the difference is <20% (these rows are left unmarked). For the rest of the categories, marked in red, the difference is larger; however it is never dramatic – the statistics derived from the study of 1000 individuals gives a very good idea of the much more precise statistics derived from 7400 individuals.

Finally, Table 7 presents the full data for the occupational distributions of the rabbinical, occupational and generic surnames. We saw on the example of the generic surnames ( Table 5) that the means obtained from the pools of about 1000 individuals provide good orientation for the actual P _i_. Therefore, we provide in separate columns the means of the three groups. It is seen that the differences are significant. For many categories, these differences continue beyond those allowed by the confidence intervals which are provided in the corresponding columns. Thus, we marked in red the rows where occupational distributions differ with 95% probability. We marked with blue the categories where the difference can be claimed with a slightly smaller probability of about 90%. The three Bhattacharyya coefficients and Hellinger distances measuring the similarity and difference of the considered three distributions are given respectively by

BC(r,g)=0.988, H(r,g)=0.1095, BC(o,g)=0.9942, H(o,g)=0.0763, BC(o,r)=0.9818, H(o,r)=0.1348,

**Table 7.** Final occupational distributions of bearers of rabbinical, occupational and generic (neither rabbinical nor occupational). The table tests the hypothesis that the occupational distributions of the groups are identical by checking the overlap of the confidence intervals. Shares P _i_(in per cent) of the i-th profession are provided for each group together with their 95% confidence intervals. The sizes of the respective pools are provided in the first row. Confidence intervals of generic surnames are significantly narrower than in other groups thanks to much larger pool of available data. Red indicates P _i_ that are different beyond the statistical error; blue indicates those that are different with high probability, e.g. would differ if we used 90% confidence interval.

Occupations	Generic surnames (n=7471)		Rabbinical surnames (n=858)		Occupational surnames (n=1056)	
	Mean %	Confidence %	Mean %	Confidence %	Mean %	Confidence %
1. Engineer	19.8	18.9<P _1_<20.7	23.2	20.4<P _1_<26	18.4	15.8<P _1_<21.0
2. Physician	7.4	6.8<P _2_<8	8.2	6.4<P _2_<10	6.3	4.7<P _2_<7.9
3. Teacher	9.8	9.1<P _3_<10.5	9.9	7.9<P _3_<11.9	9.5	7.5<P _3_<11.5
4. Bookkeeper	4.9	4.4<P _4_<5.4	4.5	3.1<P _4_<5.9	5.2	3.7<P _4_<6.7
5. Worker	18.6	17.7<P _5_<19.5	14	11.7<P _5_<16.3	23.6	20.8<P _5_<26.4
6. Creative profession	4	3.6<P _6_<4.4	4.5	3.1<P _6_<5.9	2.7	1.6<P _6_<3.8
7. Economist	3.1	2.7<P _7_<3.5	3.7	2.4<P _7_<5	3.1	1.9<P _7_<4.3
8. Head/chief officer	3.6	3.2<P _8_<4	7.5	5.7<P _8_<9.3	4.5	3.1<P _8_<5.9
9. Nurse	2.9	2.5<P _9_<3.3	2	1.1<P _9_<2.9	2.4	1.4<P _9_<3.4
10. Researcher	3.7	3.3<P _10_<4.1	5.2	3.7<P _10_<6.7	4.7	3.3<P _10_<6.1
11. Clerical worker	4.5	4<P _11_<4.9	3	1.9<P _11_<4.1	2.9	1.8<P _11_<4
12. Armed forces	3.7	3.3<P _12_<4.1	3	1.9<P _12_<4.1	3	1.9<P _12_<4.1
13. Programmer	0.8	0.6<P _13_<1	0.7	0.1<P _13_<1.3	1.6	1.2<P _13_<2.4
14. Salesman	3.5	3.1<P _14_<3.9	2.6	1.5<P _14_<3.7	2.7	1.6<P _14_<3.8
15. Businessman	2.9	2.5<P _15_<3.3	2.2	1.2<P _15_<3.2	2.6	1.5<P _15_<3.7
16. Legal profession	1.8	1.5<P _16_<2.1	0.9	0.3<P _16_<1.5	1.6	0.8<P _16_<2.4
17. Driver	1.6	1.3<P _17_<1.9	0.7	0.1<P _17_<1.3	1.7	0.9<P _17_<2.6
18. Interpreter, linguist	0.3	0.2<P _18_<0.4	0.3	P _18_<0.7	0.2	P _18_<0.5
19. Literary worker	0.6	0.4<P _19_<0.8	0.2	P _19_<0.5	0.8	0.2<P _19_<1.4
20. Pharmacist	0.9	0.7<P _20_<1.1	0.6	0.1<P _20_<1.1	1	0.3<P _20_<1.7
21. Librarian	0.4	0.3<P _21_<0.5	0.2	P _21_<0.5	0.5	P _21_<1
22. Psychologist	0.5	0.3<P _22_<0.7	0.9	0.3<P _22_<1.5	0.2	P _22_<0.5
23. Other	0.9	0.7<P _23_<1.1	1.7	0.8<P _23_<2.8	0.7	0.1<P _23_<1.3

with obvious notations (e.g. H(r,g) is the distance between rabbinical and generic surnames’ distributions). We see that occupational and rabbinical surnames’ distributions are the most different pair whereas occupational and generic surnames’ distributions are the least different pair. This does not correspond to the differences in Table 7 (colored red/blue) where the largest number of differences is between the generic and rabbinical surnames. The reason is that the pools of rabbinical and occupational surnames are smaller, which results in a larger uncertainty due to finite confidence intervals. In contrast, the overlap coefficients and the distances above are derived from the mean values only and do not reflect the magnitude of the confidence intervals.

We recall that the overlap and distance for the distributions of 1000 and 7400 generic surnames are 0.9975 and 0.05, respectively. We see, by comparison with the equation above, that these distributions are closer than distributions of different groups which is necessary for consistency. Moreover, assuming that a similar difference between distributions of 1000 and 7400 would exist for rabbinical and occupational surnames (as would be found if we had a larger pool of data), we see that the numbers are consistent with the assumption that distributions of rabbinical and occupational surnames differ from the generic surnames’ distribution and from each other.

## Discussion and conclusions

An individual’s choice of profession is determined by a multitude of genetic and environmental factors that are largely unknown. However, undoubtedly the family into which the individual is born is one of the main factors of influence. Family differences could persist for many generations via choice of partners. Indeed, marriages occur between individuals having similar social and genetic backgrounds who can preserve the differences in their offspring (see Güell *et al.*, 2015; Clark, 2015). This reproduction mechanism (which is not a literal transmission of profession from generation to generation, that was never present in our data, but rather a transmission of certain statistical preferences in occupational choices) is, however, imperfect. The differences caused by family origin gradually dissolve with time and their complete disappearance has been observed to take centuries ( Clark, 2015). In this work, we continued this direction of studies by comparison of occupational differences of the three groups of Russian-speaking Jewish families.

We observed that having a surname at which origin was a rabbi, a craftsman or neither of the above categories would create a difference of occupational preferences of the individual. For instance, some fraction of individuals having a rabbinical surname are actually the descendants of the rabbi who was at the name’s origin (and not unrelated individuals with the same surname). This results in a difference of occupational preferences of members of this group from the average preferences of the population. Since these names originated from nine to two hundred years ago then the differences in the preferences could be negligibly small today. However, previous studies, such as those reviewed by Clark (2015), indicate that the differences can still be appreciable. Our study confirmed that in fact the occupational preferences of bearers of rabbinical, occupational and generic surnames differ beyond statistical uncertainty. We remark that the studied groups themselves typically do not identify themselves as different. It can be firmly stated that most of the Russian-speaking bearers of the rabbinical surnames are either completely unaware of their name’s origin or see in it little meaning to their lives. Similar facts hold for the other groups. This differs from some cases, e.g. the case of Swedish nobility ( Clark, 2015).

Can we understand the differences provided in Table 7? Most of the differences are readily explained by low mobility, making the assumption that bearers of the names correlate appreciably with the origin of the studied surnames. Thus, the proportion of engineers among the bearers of rabbinical surnames is higher than in the other two groups. This profession demands much study and abilities for complex mind constructions that is evidently present in the profession of Jewish rabbi. The largest difference between all the three groups is in the fraction of members of the groups who are workers. The fraction for rabbinical surnames is between 0.117 and 0.163; however for generic surnames it is enclosed between 0.177 and 0.195 and for occupational surnames it is between 0.208 and 0.264. The corresponding means of occupational and rabbinical surnames differ by 1.69 times. This is a significant difference, indicating the initial inclinations of bearers of rabbinical surnames for non-worker type of activity, and conversely inclinations of craftsmen toward that kind of activity, persisted for at least two hundred years of history. These conclusions and numbers are in complete accord with those provided by Clark (2015). We find that bearers of rabbinical surnames pick the profession of heads/chief officers more than twice as often than the bearers of generic surnames. This is reasonable since rabbis led their communities. The difference from occupational surnames is somewhat smaller. Another significant difference between the groups is that bearers of both occupational and rabbinical surnames have almost identical preferences for clerical work, which are less than those of generic surnames. Inclination for clerical work would not be anticipated from a rabbi or a craftsman.

We also calculated the occupational distribution of all the surnames, i.e. general Russian-speaking Jewish families, which is of its own interest. We demonstrated that the distribution is very similar to that of bearers of generic surnames. This indicates that, despite differences between generic, rabbinical and occupational surnames being pronounced, they average out in the distribution of the general population. This is both because the deviations of the occupational preferences of bearers of rabbinical and occupational surnames from those of generic surnames are often opposite and because those groups are not as many. Thus, in our pool, rabbinical and occupational surnames constitute 20.5% (1915 individuals) of the total, where 9.2% are rabbinical surnames.

The above difference between rabbinical, occupational and generic surnames finds reasonable explanation in the personal features of rabbis and craftsmen. Similarly, we could explain the bearers of occupational surnames preference for the profession of programmer. What came as a less intuitive result is that the share of researchers among the bearers of the rabbinical surnames is larger than the average; however not that large. Further studies are needed since the difference with our data can be claimed with less than 90% probability. The bearers of the rabbinical surnames were found to have preferences for creative professions that are larger, however not much larger than the average. The bearers of occupational professions have lower preference for these professions.

Probably the most surprising of our findings is that bearers of rabbinical surnames have almost twice lower preference for legal professions than the rest of the population. It seems that since the profession of the rabbi demands the ability to learn and apply the religious law, flexibility of mind, and ability to defend sometimes opposite viewpoints, then it must be the opposite. Indeed, we checked the names of famous Russian Jewish lawyers and discovered that their surnames are overwhelmingly generic (the most famous Jewish Russian lawyer living today has an occupational surname of Reznik, which means “ritual slaughterer” ). We do not have a good explanation for this observation; however, it seems to be confirmed by the lists of prominent people of the profession in question.

Finally, the proportion of drivers among the bearers of rabbinical surnames is less than in the general Jewish population, which appears reasonable. The mean fractions of literary workers and psychologists can differ by more than four times; however, the statistical error in these rather rare groups of the population is quite large and further studies are needed. 

Here, we demonstrated the difference between the groups. The next step would be finding the actual mobility rates that characterize how fast the difference between the groups disappears. Thus, in our data we could consider the occupational distributions of each one of the four generations and compare them, where the generation is defined by an appropriate temporal period (see above and Clark, 2015). The pools that we have at our disposal are however too small for reaching definite conclusions. Therefore, the calculation of the intergenerational mobility is left for future work.

## Data availability

### Underlying data

The dataset described here cannot be shared openly due to the identifiable nature of the data (surnames, occupations, birth dates, birthplaces). Any researchers wishing to access the underlying data can contact the corresponding author ( itzhak8@gmail.com). Data will be shared under the following conditions: researchers will need to declare that they are currently undertaking similar research, that the data will not be shared with anyone other than the researcher who requested it, and it will be exclusively used for academic purposes.
