# Supplementary material for: Occupational structure of bearers of Jewish rabbinical, occupational and generic surnames
Source: F1000Res. 2020 Oct 8;9:971. Originally published 2020 Aug 11. [Version 2] doi: 10.12688/f1000research.24532.2 (PMC7721062; doi:10.12688/f1000research.24532.2)
Supplement: Peer review history

# Peer review: Reviewer response for version 2

**DOI:** 10.5256/f1000research.30040.r72725

**Published:** 2020-12-03

**Authors:** Gregory Clark

**Affiliations:** 1 Department of Economics, University of California, Davis (UC Davis), Davis, CA, USA

In the revised version the author's have clarified some points that were ambiguous or confusing in the original article.

 However, the focus of the paper on the specifics of occupational choices seems a limited use of an interesting data source. I would hope the authors would move on to consider potentially more interesting questions in future work.

Is the work clearly and accurately presented and does it cite the current literature?

Yes

If applicable, is the statistical analysis and its interpretation appropriate?

Partly

Are all the source data underlying the results available to ensure full reproducibility?

Yes

Is the study design appropriate and is the work technically sound?

Partly

Are the conclusions drawn adequately supported by the results?

Yes

Are sufficient details of methods and analysis provided to allow replication by others?

Yes

Reviewer Expertise:

Social mobility

I confirm that I have read this submission and believe that I have an appropriate level of expertise to confirm that it is of an acceptable scientific standard.

# Peer review: Reviewer response for version 2

**DOI:** 10.5256/f1000research.30040.r72726

**Published:** 2020-10-09

**Authors:** Jan Stuhler

**Affiliations:** 1 Universidad Carlos III de Madrid, Madrid, Spain

The authors have addressed my comments and I have no further concerns.

Is the work clearly and accurately presented and does it cite the current literature?

Yes

If applicable, is the statistical analysis and its interpretation appropriate?

Yes

Are all the source data underlying the results available to ensure full reproducibility?

Partly

Is the study design appropriate and is the work technically sound?

Yes

Are the conclusions drawn adequately supported by the results?

Yes

Are sufficient details of methods and analysis provided to allow replication by others?

Yes

Reviewer Expertise:

Economics, labor economics, migration, intergenerational mobility

I confirm that I have read this submission and believe that I have an appropriate level of expertise to confirm that it is of an acceptable scientific standard.

# Peer review: Reviewer response for version 1

**DOI:** 10.5256/f1000research.27060.r69225

**Published:** 2020-09-18

**Authors:** Jan Stuhler

**Affiliations:** 1 Universidad Carlos III de Madrid, Madrid, Spain

A recent development in inter-generational research is the use of names to overcome data limitations. While surnames are a rather indirect measure of family links, they are informative about distant ancestors. Compared to conventional studies based on direct family links, name-based studies can therefore capture socioeconomic differences over much longer time intervals. Studies such as Clark (2015) or Barone and Mocetti (2020) ^1^ show that status differences between surnames can persist over many centuries, which suggests that the traditional evidence based on direct parent-child links understates the extent to which advantages are transmitted from one generation to the next. Recent multi-generational studies provide some support for this view, but point to somewhat lower persistence than the surname-based studies (Santavirta and Stuhler, 2019).

 This new study by Vigdop, Norton, Rosenberg, Haguel-Spitzberg and Fouxon contribute to this literature, but adopt the surname-based approach with a twist: Rather than studying surnames per se, they compare three groups of surnames defined by their professional “bias” in the past, distinguishing names associated with rabbinical dynasties, occupational as well as generic names. This categorization exploits the distinguished position of rabbis in the Jewish population, and is comparable to the consideration of noble names in Clark (2012). The authors show that the occupational distributions of Russian-speaking Jewish families in these three groups are different, even though that in most cases, the names were inherited for many generations.

 The argument is transparently and convincingly laid out, and the main finding seems well supported by the data. I however have a number of minor comments on 1) the sample and data collection specific to this study, 2) more general methodological issues, and 3) possible extensions of this study in future research.

**Sample and data collection:**

 One wonders how representatives the sample will be, given that it was collected related to a particular educational program implemented at one particular institution. It would be useful to compare the distribution of occupations and individual characteristics to external evidence from data sources that ought to be representative, and that distinguish immigrants from the former Soviet Union. The Israeli Labor Force Survey might be such source (as for example used in Cohen-Goldner and Paserman, 2011 ^2^).

 A related issue is that respondents were are asked about their distant ancestors, such that the occupational variable might be affected by “recall bias”. It is also imaginable that such recall errors are systematic (e.g., if family folklore tends to overstate the importance of a family’s ancestors), and that this tendency varies between the groups considered in the study (e.g., if families are aware of the rabbinical origin of their name and interpret their family history accordingly). Such recall bias would tend to be more pronounced for distant ancestors. The inclusion of distant ancestors is not necessary for the analysis, and it would be useful to know if the main findings hold even when restricting attention to the subsample of the responds and more immediate ancestors.

**More general m** **ethodological issues:**

 One of the main criticism against the name-based approach is that it measures status persistence on the group (surname) level rather than individual-level persistence. The group-level perspective becomes problematic if the group definitions correlate with third factors that itself have implications for socioeconomic status and intergenerational mobility. In particular, surnames may differ systematically with race or location. It would therefore be interesting to test whether families in the three groups came from systematically different regions in the Former Soviet Union (e.g., by coding regions in terms of their geographic location, their population density, or other regional characteristics).

 At first, the splitting of the total sample into blocks of hundreds seems a little odd. The best predictor of the population mean is the sample mean (e.g., the last column in Table 2), so it is unusual to split the sample into subsamples to do separate analysis within each of these subsamples (e.g., the other columns in Table 2). Of course, the purpose of this procedure is to illustrate how sensitive the results are to sample size, given that we have much fewer rabbinical and occupational than general surnames. However, it does not become sufficiently clear why (i) we cannot just estimate standard errors for the occupational shares, and the difference in occupational shares between surname groups, and (ii) in which way splitting the total sample into separate blocks can be informative about the random sampling assumption. To motivate that early on would be useful, because the discussion of this issue in the current paper version takes much space and becomes fairly technical.

 In the final part of the manuscript it becomes clear why sampling uncertainty is such an important issue – the Bhattacharyya coefficient and the Hellinger distance do not account for sampling uncertainty, so tends to indicate that occupational distributions are more distant when the sample size is small. This caveat could be mentioned earlier on. As such, the Bhattacharyya and Hellinger measures cannot fully serve their intended purpose. The authors address this issue by assessing the sample fractions and corresponding confidence intervals for each of the professions in Table 7. These “case studies” illustrate that the difference in occupational distributions is indeed systematic, and not just a statistical artifact from sampling error. However, this evidence is not integrated with the summary measures, and for future research it would be useful to study whether we can find a measure of occupational distance that already accounts for sampling error.

 Minor presentational issues: ****In the abstract, it could be briefly noted what database you are referring to (e.g., that the data is self-collected). In Table 7, it could be clarified if you formally test the hypothesis that the difference in population means between the groups is zero, or simply compare whether the confidence intervals are overlapping.

**P** **ossible extensions** **and future research:**

 A potential fruitful avenue for future research would be the application of occupational prestige scores that rank occupations according to their socioeconomic status. Of course, these type of rankings are not without issues – the standing of an occupation may differ between countries, or vary over time. See Yuchtman and Fishelson (1972) ^3^, for a consideration of these issues in the Israelian context. However, such issues would be less problematic if our main focus is on the comparison between groups, and errors in the rankings affect the groups similarly. By applying occupational scores, we transform the categorical into ordered data, which would allow for a number of interesting extensions in future research. Most importantly, it would allow us to change focus from group differences in the occupational structure as such (in any direction) to systematic differences in socioeconomic status, and therefore intergenerational mobility.

 Once occupational scores are matched to the data, we can also apply the type of name-based estimators that are increasingly used in the intergenerational literature (for an overview, see Santavirta and Stuhler, 2020). For example, we can study to what extent surnames or the rabbinical/occupational/general categorization can predict socioeconomic success, using the approach by Güell et al. 2015. The other widely used approach is the so-called grouping estimator, to measure how quickly the difference between groups regress to the mean. While the estimator depends on sample size, it performs better in the type of data structure that we face here, in which complete lineages are sampled. In particular, it would be interesting to understand if the apparent status differences between rabbinical, occupational and general surnames have remained stable, or reduced over the generations covered by your sample.

Is the work clearly and accurately presented and does it cite the current literature?

Yes

If applicable, is the statistical analysis and its interpretation appropriate?

Yes

Are all the source data underlying the results available to ensure full reproducibility?

Partly

Is the study design appropriate and is the work technically sound?

Yes

Are the conclusions drawn adequately supported by the results?

Yes

Are sufficient details of methods and analysis provided to allow replication by others?

Yes

Reviewer Expertise:

Economics, labor economics, migration, intergenerational mobility

I confirm that I have read this submission and believe that I have an appropriate level of expertise to confirm that it is of an acceptable scientific standard.

## References

[1] : Intergenerational Mobility in the Very Long Run: Florence 1427-2011. *SSRN Electronic Journal*.2016; 10.2139/ssrn.2856359 10.2139/ssrn.2856359

[2] : The dynamic impact of immigration on natives' labor market outcomes: Evidence from Israel. *European Economic Review*.2011;55(8) : 10.1016/j.euroecorev.2011.05.002 1027-1045 10.1016/j.euroecorev.2011.05.002

[3] : Some Problems in the Study of Occupational Prestige with an Illustration from Israel. *The British Journal of Sociology*.1972;23(2) : 10.2307/589107 10.2307/589107

# Response

**Published:** 2020-10-02

**Authors:** Itzhak Fouxon

**Affiliations:** Yonsei University, South Korea

**Review by Prof. Jan Stuhler**

A recent development in inter-generational research is the use of names to overcome data limitations. While surnames are a rather indirect measure of family links, they are informative about distant ancestors. Compared to conventional studies based on direct family links, name-based studies can therefore capture socioeconomic differences over much longer time intervals. Studies such as Clark (2015) or Barone and Mocetti (2020)1 show that status differences between surnames can persist over many centuries, which suggests that the traditional evidence based on direct parent-child links understates the extent to which advantages are transmitted from one generation to the next. Recent multi-generational studies provide some support for this view, but point to somewhat lower persistence than the surname-based studies (Santavirta and Stuhler, 2019).

This new study by Vigdop, Norton, Rosenberg, Haguel-Spitzberg and Fouxon contribute to this literature, but adopt the surname-based approach with a twist: Rather than studying surnames per se, they compare three groups of surnames defined by their professional “bias” in the past, distinguishing names associated with rabbinical dynasties, occupational as well as generic names. This categorization exploits the distinguished position of rabbis in the Jewish population, and is comparable to the consideration of noble names in Clark (2012). The authors show that the occupational distributions of Russian-speaking Jewish families in these three groups are different, even though that in most cases, the names were inherited for many generations.

The argument is transparently and convincingly laid out, and the main finding seems well supported by the data. I however have a number of minor comments on 1) the sample and data collection specific to this study, 2) more general methodological issues, and 3) possible extensions of this study in future research.

**Response**: We thank Prof. Jan Stuhler for kind opinion of our work. We hope that we could incorporate the minor comments in the revised version, please see below.

**Review**: Sample and data collection:

One wonders how representatives the sample will be, given that it was collected related to a particular educational program implemented at one particular institution. It would be useful to compare the distribution of occupations and individual characteristics to external evidence from data sources that ought to be representative, and that distinguish immigrants from the former Soviet Union. The Israeli Labor Force Survey might be such source (as for example used in Cohen-Goldner and Paserman, 20112).

**Response**: The participation in our program was obligatory for all Russian-speaking participants of a larger program which was held for many years and in which over all about one million young Jews took part. This larger program is extremely inclusive and the only bias that it could introduce is that all participants had some degree of affiliation with the Jewish people. Hence, strictly speaking, our findings are confined to this subgroup of carriers of rabbinical, occupational and generic surnames. The Labor Force Survey obviously would have a similar bias and we do not see how we could perform a study beyond the above limitation. It seems to us that this bias is not so restrictive since it roughly corresponds to the original statement that the study was performed for Russian-speaking Jewish families. We have introduced in the text an explicit statement  "Our program was obligatory for participants of a larger, very inclusive program so that to the best of our knowledge the only bias in the sample was some degree of affiliation with the Jewish people."  We hope that this resolves the issue. 

**Review**: A related issue is that respondents were are asked about their distant ancestors, such that the occupational variable might be affected by “recall bias”. It is also imaginable that such recall errors are systematic (e.g., if family folklore tends to overstate the importance of a family’s ancestors), and that this tendency varies between the groups considered in the study (e.g., if families are aware of the rabbinical origin of their name and interpret their family history accordingly). Such recall bias would tend to be more pronounced for distant ancestors. The inclusion of distant ancestors is not necessary for the analysis, and it would be useful to know if the main findings hold even when restricting attention to the subsample of the responds and more immediate ancestors.

**Response**:  We agree that this could be an issue. Unfortunately cutting the data would result in statistically insignificant sample. We believe though that recall bias is not high and the mistake in reporting the profession of grandparents can be neglected (The reported data on the ggparents boiled down to parents' data on their gparents. The participants could tell that they do not know as they did occasionally). 

Moreover the recall bias, in this case, could act in the direction of erasing differences between the groups rather than amplifying them. Indeed, the Soviet totalitarian regime made any professions of ancestors other than peasants and workers very problematic. Cutting the heads which were above the crowd was more than usual. For instance wood merchants would be reported as wood experts, the profession of rabbi would be suppressed as much as possible and so forth. Hence the recall bias almost certainly acted in the opposite direction of erasing all differences between the USSR citizens. 

**Review**: More general methodological issues:

One of the main criticism against the name-based approach is that it measures status persistence on the group (surname) level rather than individual-level persistence. The group-level perspective becomes problematic if the group definitions correlate with third factors that itself have implications for socioeconomic status and intergenerational mobility. In particular, surnames may differ systematically with race or location. It would therefore be interesting to test whether families in the three groups came from systematically different regions in the Former Soviet Union (e.g., by coding regions in terms of their geographic location, their population density, or other regional characteristics).

**Response**: we thought of this factor to which we devoted this paragraph that probably went overlooked: 

"The birthplaces were scattered all over the territory of the FSU. Jewish families have a long tradition of studying and those who would want to acquire education would typically receive such an opportunity. In other words, with a good approximation, an individual born into a Jewish family of the FSU would have an equal opportunity for getting that or another profession irrespective of birthplace. Therefore, we disregarded the geographical factor in our study."

**Review**: At first, the splitting of the total sample into blocks of hundreds seems a little odd. The best predictor of the population mean is the sample mean (e.g., the last column in Table 2), so it is unusual to split the sample into subsamples to do separate analysis within each of these subsamples (e.g., the other columns in Table 2). Of course, the purpose of this procedure is to illustrate how sensitive the results are to sample size, given that we have much fewer rabbinical and occupational than general surnames. However, it does not become sufficiently clear why (i) we cannot just estimate standard errors for the occupational shares, and the difference in occupational shares between surname groups, and (ii) in which way splitting the total sample into separate blocks can be informative about the random sampling assumption. To motivate that early on would be useful, because the discussion of this issue in the current paper version takes much space and becomes fairly technical.

**Response**:  We introduced in the revised version the early stage explanation: "In contrast with individual data which is not randomly sampled, the blocks can be be considered as a result of random sampling where a hundred was taken from the population and then, independently, another hundred and so forth, see below. Moreover separation into blocks demonstrates what we can anticipate to see if we pick 100 members from the considered population: " We hope that this early explanation helps the reader. 

**Review**:  In the final part of the manuscript it becomes clear why sampling uncertainty is such an important issue – the Bhattacharyya coefficient and the Hellinger distance do not account for sampling uncertainty, so tends to indicate that occupational distributions are more distant when the sample size is small. This caveat could be mentioned earlier on. As such, the Bhattacharyya and Hellinger measures cannot fully serve their intended purpose. The authors address this issue by assessing the sample fractions and corresponding confidence intervals for each of the professions in Table 7. These “case studies” illustrate that the difference in occupational distributions is indeed systematic, and not just a statistical artifact from sampling error. However, this evidence is not integrated with the summary measures, and for future research it would be useful to study whether we can find a measure of occupational distance that already accounts for sampling error.

**Response**:  We introduced in the revised version the early stage explanation: "We will see below that on their own the distances do not allow the distributions’ comparison, however in conjunction with statistical analysis they become useful." The problem of introducing a distance accounting for sampling error seems to us very difficult indeed. 

**Review**:  Minor presentational issues: In the abstract, it could be briefly noted what database you are referring to (e.g., that the data is self-collected). In Table 7, it could be clarified if you formally test the hypothesis that the difference in population means between the groups is zero, or simply compare whether the confidence intervals are overlapping.

**Response**:  We introduced in the abstract "self-collected". We introduced in the table's caption the sentence "The table tests the hypothesis that the occupational distributions of the groups are identical by checking the overlap of the confidence intervals."  

**Review**:    

Possible extensions and future research:

A potential fruitful avenue for future research would be the application of occupational prestige scores that rank occupations according to their socioeconomic status. Of course, these type of rankings are not without issues – the standing of an occupation may differ between countries, or vary over time. See Yuchtman and Fishelson (1972)3, for a consideration of these issues in the Israelian context. However, such issues would be less problematic if our main focus is on the comparison between groups, and errors in the rankings affect the groups similarly. By applying occupational scores, we transform the categorical into ordered data, which would allow for a number of interesting extensions in future research. Most importantly, it would allow us to change focus from group differences in the occupational structure as such (in any direction) to systematic differences in socioeconomic status, and therefore intergenerational mobility.

Once occupational scores are matched to the data, we can also apply the type of name-based estimators that are increasingly used in the intergenerational literature (for an overview, see Santavirta and Stuhler, 2020). For example, we can study to what extent surnames or the rabbinical/occupational/general categorization can predict socioeconomic success, using the approach by Güell et al. 2015. The other widely used approach is the so-called grouping estimator, to measure how quickly the difference between groups regress to the mean. While the estimator depends on sample size, it performs better in the type of data structure that we face here, in which complete lineages are sampled. In particular, it would be interesting to understand if the apparent status differences between rabbinical, occupational and general surnames have remained stable, or reduced over the generations covered by your sample.

**Response**:  We agree with the referee that these are good directions for future research. We would like to thank Prof. Stuhler for devoting time to our work and providing thoughtful comments.

# Peer review: Reviewer response for version 1

**DOI:** 10.5256/f1000research.27060.r69224

**Published:** 2020-08-28

**Authors:** Gregory Clark

**Affiliations:** 1 Department of Economics, University of California, Davis (UC Davis), Davis, CA, USA

This article seeks to detect whether there is occupational persistence among Russian Jewish immigrants to Israel and their ancestors with the occupational types implied by their surnames - rabbinical, craft occupations, or nondescript. The authors find that the occupational pattern of the inheritors of rabbinical surnames does differ still from the inheritors of nondescript surnames, while the craft surname inheritors show less difference.

 A strength of the article is the database the authors have collected by surveying Russian Jewish immigrants to Israel of up to 4 generations of occupations in each family. This database has occupational information on more than 9,000 linked individuals.

 The article has limitations however. First the focus on occupational structure makes the problem one of high dimensions statistically, with limitations on the statistical significance of results, and the results hard to evaluate. It is hard, for example, to compare what is happening with this group compared to other populations. Simpler questions could be addressed with this data which would yield statistically much stronger results. For example, we can assign a status score to each occupation by looking at average earnings by occupation now in Israel. Then we could calculate this average status score across each of 4 generations. We could then estimate how quickly across generations status was regressing to the mean in this population. And occupational persistence may be just a variety of status persistence if occupations vary by status.

 I am most familiar with English data, and here the intergenerational persistence of status is much stronger than the persistence of occupational type. Priests have sons who are almost equally likely to be army officers, lawyers, doctors, civil servants, engineers, and university researchers.

 A second limitation of the article is that while the nondescript surnames constitute about 80% of the sample, for some reason only a subset of these cases are used in much of the analysis.  It was never clearly explained why this was the case. If the authors have the data then I do not see why this limitation would be imposed.

 A third limitation is that while the data covers 4 generations, in the analysis it was all lumped into one pool, losing all the temporal information. I think this was because it was necessary because of the high dimension statistical problem to aggregate the data to obtain results significant at conventional significance levels. But why introduce this feature of the data if no use is made of it?

 A fourth limitation is that the statement that these surnames were at least 200 years old in origin was never justified. How do the authors know that?

 A suggestion for further work would be to look at other surname types.  In 19th century Russia the surname ending ..ski was elite, while the endings ..ov, ..in, and ..ev were lower class. Do any of these immigrants have such endings and do they show any distinctiveness?

Is the work clearly and accurately presented and does it cite the current literature?

Yes

If applicable, is the statistical analysis and its interpretation appropriate?

Partly

Are all the source data underlying the results available to ensure full reproducibility?

Yes

Is the study design appropriate and is the work technically sound?

Partly

Are the conclusions drawn adequately supported by the results?

Yes

Are sufficient details of methods and analysis provided to allow replication by others?

Yes

Reviewer Expertise:

social mobility

I confirm that I have read this submission and believe that I have an appropriate level of expertise to confirm that it is of an acceptable scientific standard, however I have significant reservations, as outlined above.

# Response

**Published:** 2020-10-02

**Authors:** Itzhak Fouxon

**Affiliations:** Yonsei University, South Korea

**Review by Prof. Gregory Clark**

 This article seeks to detect whether there is occupational persistence among Russian Jewish immigrants to Israel and their ancestors with the occupational types implied by their surnames - rabbinical, craft occupations, or nondescript. The authors find that the occupational pattern of the inheritors of rabbinical surnames does differ still from the inheritors of nondescript surnames, while the craft surname inheritors show less difference.

**Response**: the description is accurate with sole reservation that the participants were visitors of Israel and not immigrants. The misunderstanding probably stems from reading   

 "The individuals were Russian-speaking Jewish family members residing in the Former Soviet Union (FSU). They voluntarily provided genealogical data for the program via online forms that were sent to them before their arrival in Tel Aviv (see section Data collection)...."

 which does allow for misunderstanding. We introduced the sentence "Upon the completion of the educational program, the participants returned to their home countries." for clarification. 

**Review**: A strength of the article is the database the authors have collected by surveying Russian Jewish immigrants to Israel of up to 4 generations of occupations in each family. This database has occupational information on more than 9,000 linked individuals.

**Response**: We thank the reviewer for kind opinion of our efforts in data collection. 

**Review**: The article has limitations however. First the focus on occupational structure makes the problem one of high dimensions statistically, with limitations on the statistical significance of results, and the results hard to evaluate. 

**Response**: It is true that having a high dimensional statistics made our task much harder. We needed to collect data on a large number of participants which took us four years. We stopped the data collection when we saw that the results are already statistically significant. We had the purpose in this paper to show that with 95% probability the statistics of different groups are different. We believe that this observation deserves to be reported. There is much more to be learnt from the data as the referee remarks below. 

**Review**: It is hard, for example, to compare what is happening with this group compared to other populations. 

**Response**: We did in fact check that we can make a comparison with non-Jewish participants of the program (who belong to Jewish families by marriage). However doing all this work in one paper would be too much (this work took a lot of time) and we left the detailed study for future work. It is true that for comparison with yet other populations would demand a different approach. 

**Review**: Simpler questions could be addressed with this data which would yield statistically much stronger results. For example, we can assign a status score to each occupation by looking at average earnings by occupation now in Israel. Then we could calculate this average status score across each of 4 generations. We could then estimate how quickly across generations status was regressing to the mean in this population. And occupational persistence may be just a variety of status persistence if occupations vary by status.

**Response**: We thank the referee for an excellent idea for future work. In fact, we struggled to get statistically significant results for separate generations, we simply would need a much larger database. Using the status score would allow us to get these results, it seems. We could estimate regression to the mean and mobility. This would make our work a contribution to the study of mobility, which is a traditional object of much interest.

 At the same time, our data gives detailed occupational structure of these generations as a whole i.e. of Russian Jewry during the twentieth century. This object does not give us details of generational distribution, however gives us details on occupational distribution, albeit coarse-grained over four generations. The occupational distribution has its own interest, as we hope the referee would agree. Thus we could quantify if members of rabbinical families are strongly overrepresented in research work and study other questions. Our opinion is that both studies are valuable and "much stronger results" still does not mean that our work does not present interest.

**Review**: I am most familiar with English data, and here the intergenerational persistence of status is much stronger than the persistence of occupational type. Priests have sons who are almost equally likely to be army officers, lawyers, doctors, civil servants, engineers, and university researchers.

**Response**: We are aware of the outstanding research by the referee in this direction. If the conclusions of our study can be applied to English data then they tell that spreading of professions of descendants of priests will produce a distribution different from that of descendants of craftsmen.

**Review**: A second limitation of the article is that while the nondescript surnames constitute about 80% of the sample, for some reason only a subset of these cases are used in much of the analysis.  It was never clearly explained why this was the case. If the authors have the data then I do not see why this limitation would be imposed.

**Response**: We did use the full data e.g. table 7, providing the total distributions, states that data on 7471 individuals with nondescript surnames was used. However in the course of the discussion we compare the distributions for 7471 and 1000 individuals with nondescript surnames in order to show the reader what the difference could be for rabbinical and occupational surnames where we have data on 1000 however not on 7471 individuals. For stressing this point we introduced in the caption of Table 7 the sentence "Confidence intervals of generic surnames are significantly narrower than in other groups thanks to much larger pool of available data."

**Review**: A third limitation is that while the data covers 4 generations, in the analysis it was all lumped into one pool, losing all the temporal information. I think this was because it was necessary because of the high dimension statistical problem to aggregate the data to obtain results significant at conventional significance levels. 

**Response**: It is the true reason. We include in the revised version an explicit statement in the sentence:"We did not perform separate study of different cohorts since the data available for them would not be statistically significant."

**Review**: But why introduce this feature of the data if no use is made of it?

**Response**: Our main target in the paper is showing the groups' difference. This could occur because different generations could be present differently in these groups. For instance by pure accident we could have much more representatives of early generations for rabbinical surnames than for occupational ones. Then a possible reason for the difference could be time dependence of the average occupational distribution in the society. Thus we write that "For adequate comparison of the groups, we must have roughly the same share of each group born in each of the considered generations." The data processing would not change if we omitted the feature, however the implications would change should we have found a strong difference of generations present in the groups. 

**Review**: A fourth limitation is that the statement that these surnames were at least 200 years old in origin was never justified. How do the authors know that?

**Response**: Surnames' adoption by Jews was a process that took many centuries. However the official laws ordered universal adoption of hereditary family names everywhere Jews, considered in this study resided, at about 1800. Strictly speaking, inaccuracy of law enforcement in Russia, introduced some exceptions to this rule where some Jews could avoid the census and the names' adoption for some time. However these exceptions are statistically negligible. We introduced the sentence "Formation of Jewish surnames with few exceptions finished by the beginning of the 19th century, see e. g. Beider, 2008." that provides a reference with exposition of the history of the surnames' adoption by Jews. We thank the reviewer for this comment, this had to be told.

**Review**: A suggestion for further work would be to look at other surname types.  In 19th century Russia the surname ending ..ski was elite, while the endings ..ov, ..in, and ..ev were lower class. Do any of these immigrants have such endings and do they show any distinctiveness?

**Response**: These endings are atypical endings for the Jewish surnames and rather belong to Russian surnames. We'd be glad to make this study however we do not have these data at the moment. 

**Review**: I confirm that I have read this submission and believe that I have an appropriate level of expertise to confirm that it is of an acceptable scientific standard, however I have significant reservations, as outlined above.

**Response**: We cordially thank the Referee for in-depth study of the paper and constructive critics and comments. We hope that our revisions in the text and responses above resolve the significant reservations by the referee and the present version of the paper can be approved.
